# Fire Properties of Paper Sheets Made of Cellulose Fibers Treated with Various Retardants

**DOI:** 10.3390/ma17133074

**Published:** 2024-06-22

**Authors:** Zuzanna Szubert, Bartłomiej Mazela, Karolina Tomkowiak, Wojciech Grześkowiak

**Affiliations:** 1 Faculty of Forestry and Wood Technology, Poznań University of Life Sciences, Wojska Polskiego 28, 60-637 Poznań, Poland; 2 Przedsiębiorstwo Wielobranżowe Sp. z o. o., Polna 1, 64-316 Michorzewo, Poland

**Keywords:** cellulose, paper products, molded fibers, fire retardants, mass loss calorimeter, mini fire tube

## Abstract

This article presents the results of flame-retardancy tests conducted on cellulose sheets produced using a Rapid Köthen apparatus treated with retardants. The agents used were potassium carbonate (PC) K_2_CO_3_ (concentrations of 20; 33.3; and 50% wt/wt), monoammonium phosphate (MAP) NH_4_H_2_PO_4_ (concentrations of 35% wt/wt), diammonium phosphate (DAP) (NH_4_)_2_HPO_4_ (concentrations of 42.9% wt/wt), and bisguanidal phosphate (FOS) C_2_H_10_N_6_ (concentrations of 22.5% wt/wt). The agents were used to improve Kraft cellulose-based sheets’ flame-retardant properties and compare their performances. As part of the study, the flammability of the materials was determined by the following methods: an oxygen index (OI) test, a mass loss calorimeter (MLC) test, and a mini fire tube (MFT) test. All formulations showed an increase in flame retardancy compared to the control test. All protected samples were non-flammable for OI determinations, and DAP-protected samples showed the highest OI index. For the MLC test, DAP-protected and MAP-protected samples showed the best heat-release rate (HRR), total heat release (THR), and average heat-release rate (ARHE) (samples did not ignite for 600 s). In the MFT test, all treated samples had comparably reduced weight loss. The best parameter was achieved for MAP and DAP (15% weight loss).

## 1. Introduction

In recent years, paper’s flammability and resistance to fire have become the subject of increased interest and research. The increased need for fire-resistant materials has made the issue of paper flammability crucial, especially for industries where paper is commonly used. Understanding and controlling the flammability of paper is essential for ensuring safety and minimizing the risks associated with fires, which are particularly important for preventing potentially catastrophic consequences [1].

Fireproofing agents can be divided into penetrating agents (impregnants), surface agents (varnishes, paints), or agents that are added directly to the mass [2,3,4].

Various methods have been developed for enhancing the flame retardancy of cellulose, which can be divided into several main categories: physical, chemical, and mechanical. Physical fire-protection methods involve creating protective layers on the surface of the cellulose that prevent the penetration of heat and oxygen, which are essential for sustaining combustion. Chemical methods focus on introducing chemical compounds into the cellulose’s structure that release substances during combustion to inhibit the burning process. These compounds can act by lowering the ignition temperature, releasing non-flammable gases, or forming protective barriers in the form of char. Mechanical methods involve structural changes in the cellulose, such as surface modifications, which make it more difficult for the fire to spread [5].

There are a number of measures that help to reduce the flammability of materials. Phosphorus-containing flame retardants work by forming an intumescent layer. This accumulates on the surface and forms a protective barrier that inhibits flames. Phosphorus flame retardants can not only promote the formation of a carbon layer to isolate the exchange of matter and energy but also produce free radicals that are responsible for extinguishing the thermal decomposition reaction [6].

The flame-retardant properties of phosphorus–nitrogen compounds, such as monoammonium phosphate and diammonium phosphate, stem from their dual action in both the condensed and gas phases [2,7]. During combustion, these flame retardants decompose into phosphoric acid and polyphosphoric acid, creating a non-volatile protective layer on the substrate material’s surface, thereby impeding heat transmission. Additionally, these compounds have the potential to release ammonia, nitrogen, water vapor, and other non-flammable gases, which dilute the combustible gases and lower the material’s surface temperature by absorbing heat. This effectively prevents the material from igniting [8,9].

As for the mechanisms of action of the fireproofing agents themselves, a distinction can be made between the coating mechanism, which consists of stopping the transport of heat by forming a non-flammable coating before the material reaches the temperature of thermal decomposition and pyrolysis. This mechanism slows the emission of gaseous combustion products [5,10].

The thermal mechanism of flame retardants is based on the manipulation of heat transfer during the combustion processes of materials, aiming to control the temperature of the material and delay its ignition. These agents react to reduce heat transfer into the material. There are various methods by which this action is achieved. The carbon layer formed on the surface of the material and the absorption of heat by the flame retardants are two ways to manipulate heat transfer. In addition, some of these agents can also lower the thermal conductivity of the material, effectively reducing heat transfer deep into the material [5].

The other mechanism is the gas mechanism, which involves the release of gases from the flame retardants. The gases emitted are either non-flammable gases or gases that inhibit combustion reactions. The gas mechanism is effective because it works on several levels simultaneously. It dilutes flammable gases, lowers the temperature, and chemically inhibits combustion reactions. The gases released perform several important functions, such as diluting the flammable gases that result from the pyrolysis of the material, cooling the surface of the material, and the ability to inhibit combustion reactions [10].

The chemical mechanism of flame retardants involves the introduction of chemicals into the structure of the material that release substances that inhibit combustion processes during combustion. These substances can act in several ways, such as lowering the ignition temperature, releasing non-combustible gases, or forming non-combustible carbon barriers. Through this mechanism, the material becomes less susceptible to fire and more difficult to ignite [5,10,11]. Most fire retardants work by combining several mechanisms at once, demonstrating high effectiveness.

Ghanadpour et al. (2015) discovered that adding phosphate groups to cellulose nanofibers using diammonium phosphate and urea as flame retardants greatly enhances their ability to inhibit combustion [2].

Grześkowiak (2012) tested wood treated with urea alone and mixtures thereof with potassium carbonate [8]. The results showed that the addition of potassium carbonate positively impacted the reduction of heat release and the heat of combustion while also increasing the ignition time. Potassium carbonate caused a shift in the initial pyrolysis temperature and the temperature of total cellulose decomposition to a lower value [12].

Guanidine salts are a type of flame retardant that does not contain halogens. Upon heating, guanidine salts release NH_3_ or CO_2_ to form a non-flammable charred layer [13,14]. As per the literature, guanidine salts have the potential to decrease the flammability of paper and its combustion rate. In addition to this, it may also lower the amount of toxic gases and smoke produced during paper combustion. These properties are significant from a fire-safety standpoint [14,15]. 

The work aimed to evaluate the effect of substances in the form of potassium carbonate, monoammonium phosphate, diammonium phosphate, and bisguanidine phosphate on the fire properties of cellulose sheets. The research method, and in particular the cellulose-sheets production process, was designed to simulate the conditions prevailing in molded-fibers technology. The scope of work included preparing cellulose sheets and applying potential flame retardants to them. The fire behavior of the cellulose sheets was determined using three experiments: mini fire tube, mass loss calorimeter, and oxygen index.

## 2. Materials and Methods

### 2.1. Materials

This study used Kraft pulp fibers (Guaíba, commercial form) made from 100% eucalyptus (ECF bleaching process) to produce the samples. The brightness was 89 + % (ISO 2470 [16]), dirt count ≤ 2.5 mm^2^/kg a.d. (ISO 5350-2 [17]), viscosity ≥ 700 mL/g (ISO 5351 [18]), and pH ≥ 5.0 (ISO 6588-1 [19]). The reagents used were potassium carbonate (CAS: 584-08-7) in concentrations of 20%, 33.3%, and 50%; bisguanidine phosphate at 22.5% (CAS: 5423-23-4); diammonium phosphate (CAS: 7783-28-0) at 42.9%, and monoammonium phosphate (CAS: 7722-76-1) at 35%. Deionized water was used in the production of all sample variants.

### 2.2. Preparation of Cellulose Sheets

Cellulose sheets (diameter = 200 ± 0.1 mm, thickness = 0.5 ± 0.09 mm) were produced using a Rapid Köthen sheet-forming machine (Labor-Meks, Łódź, Poland). This process entails utilizing a cylindric container equipped with a sieve at its base. The container was filled with a mixture of pulp and water, which was subsequently homogenized by stirring it within the container. Following this, a valve at the container’s bottom was opened, allowing the water to drain and leaving the pulp on the sieve. The resulting wet pulp sheet was then transferred to a dryer with the aid of a carrier board. Before drying, a cover sheet was placed on top of the wet pulp sheet. The sheet was then dried between the carrier board and the cover sheet inside the dryer. Once the sheet was thoroughly dry, the carrier board and paper cover sheet were removed and conditioned before testing. Twenty Kraft pulp discs with a grammage of 166.67 g/m^2^ of paper were produced [20].

### 2.3. Impregnation and Pressing of the Sheets

The impregnation of the samples was carried out by spraying the cellulose sheets. The consumption norm for a 0.03 m^2^ disc was 4.5–6 g. PC was applied to the sheets at concentrations of 20% (25 g/100 g water), 33.3% (50 g/100 g water), and 50% (100 g/100 g water), and FOS with a concentration of 22.5% (29 g/100 g water). DAP with a concentration of 42.9% (75 g/100 g water) was achieved by dissolving the substance in water at a temp. 55 °C; MAP with a concentration of 35% (53.9 g/100 g water) was achieved by dissolving the substance in water at 49 °C. The amount of application was constantly monitored using an analytical balance (Table 1). Pressing of the sheets with the applied impregnant was carried out in a hydraulic press at 220 °C, with a load of 1 t in 40 s. The sheets were separated by a mesh. The sheet production process presented was intended to simulate on a laboratory scale how cellulose pulp is molded under industrial conditions using molding-fibers technology [21]. In this research, the following variants were produced: untreated paper (labeled as CONTROL); sample treated with 20% PC (labeled as 20PC); sample treated with 33.3% PC (labeled as 33.3PC); sample treated with 50% PC (labeled as 50PC); sample treated with 100% FOS (labeled as FOS); sample treated with 35% MAP (labeled as MAP); and sample treated with 42.9% DAP (labeled as DAP).

### 2.4. Flammability Properties Assessment

#### 2.4.1. Mini Fire Tube

**Figure 1:**
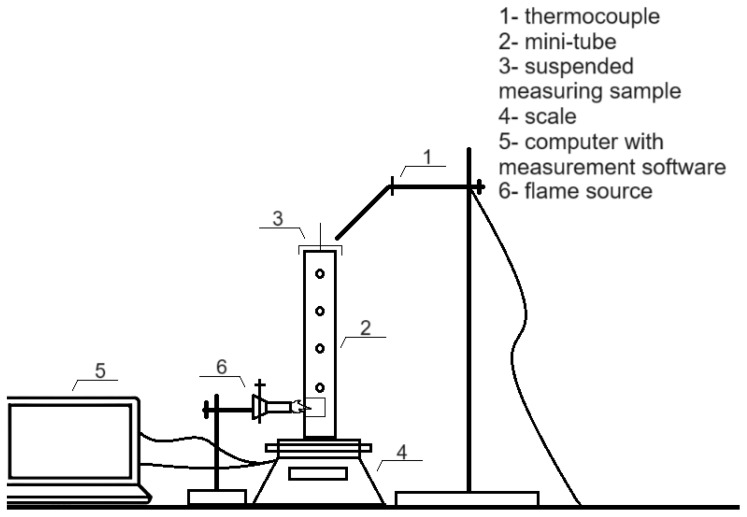
Scheme of measurement apparatus for MFT.

The MFT method was adapted and modified based on the ASTM E69 (2022) standards [22]. The analytical balance held an aluminum profile tube that was 20 cm in length and 20 mm in diameter (Figure 1). The tube was heated with a gas burner placed on a trestle, which produced a 1 cm high flame. The temperatures of the exhaust gases were measured using a k-type thermocouple with a temperature range of 50–1200 °C [14]. The MFT measurement was performed using at least ten repetitions of each sample, which were 10 ± 1 mm in length, 100 ± 1.5 mm in width, and an initial weight of 0.35 ± 0.05 g.

#### 2.4.2. Mass Loss Calorimeter

The MLC (FTT—Fire Testing Technology—East Grinstead, UK) test was used to evaluate important fire-retardancy parameters, such as peak heat-release rate (PHRR), heat-release rate, total heat released, and mean value of effective heat of combustion (EHC), as a function of time. The test was carried out using three sample repetitions (100 × 100 × 0.5 mm). The heat flux of the cone used was 35 kW/m^2^, and the sample distance from the cone was 25 mm. The MLC test was conducted by ISO 13927 [23].

#### 2.4.3. Oxygen Index

OI measures the concentration of oxygen needed for the combustion of organic materials after ignition. The OI measurement method has been standardized nationally (PN-76 C-89020 [24]) and internationally (ISO 4589 [25]). The test sample is exposed to a mixture of oxygen and nitrogen until a uniform burning flame is achieved. The oxygen concentration at that point is the oxygen index [3,26,27]. Janowska et al. (2007) consider materials with an oxygen index of less than or equal to 21% as flammable, while materials with an oxygen index greater than 21% are considered fireproof [28]. On the other hand, Gilman (1999) considers materials with an oxygen index between 21–28% as fireproof and those with an oxygen index equal to or greater than 28% as non-combustible [29,30].

## 3. Results and Discussion

### 3.1. Fire Properties

#### 3.1.1. Mini Fire Tube

According to the percentage of mass loss (ML) of the samples obtained from the MFT results (Table 2), the untreated CONTROL cellulose sheet showed an ML of 99%. The addition of retardants to the samples reduced their mass loss. Applying PC to the samples at concentrations of 20%, 33.3%, and 50%, respectively, showed a significant reduction in ML. For a concentration of 20%, the ML was 29%, and for 33.3% and 50% concentrations, the ML was 19% and 24%, respectively. No correlation could be indicated between the concentrations themselves. The reason for the effect of reduced weight loss is probably due to the formation of a barrier layer for charcoal and the release of non-flammable gas during combustion, which significantly delayed the endothermic depolymerization of cellulose glucopyranose units [8,12,13,31,32]. 

The same ML of 15% was observed when MAP and DAP were used. This may have been influenced by the ability of these compounds to stabilize the combustion process and reduce toxic emissions [33]. FOS showed a mass loss of 18%. According to the literature, depending on the application and concentration, FOS can reduce the flammability of paper and the combustion rate. In addition, it can reduce the amount of smoke and toxic gases produced during paper combustion, which can be important from a fire-safety perspective [14,15].

It is worth noting that the maximum temperature of the exhaust gases (Table 2) was reduced for each agent used. In the case of both phosphate and potassium carbonate, this effect can be explained by the theory of non-combustible gas emissions. This means that the release of non-combustible volatile products formed as a result of the formation of the protective barrier increases significantly in the modified samples [34,35,36].

#### 3.1.2. Mass Loss Calorimeter

**Figure 2:**
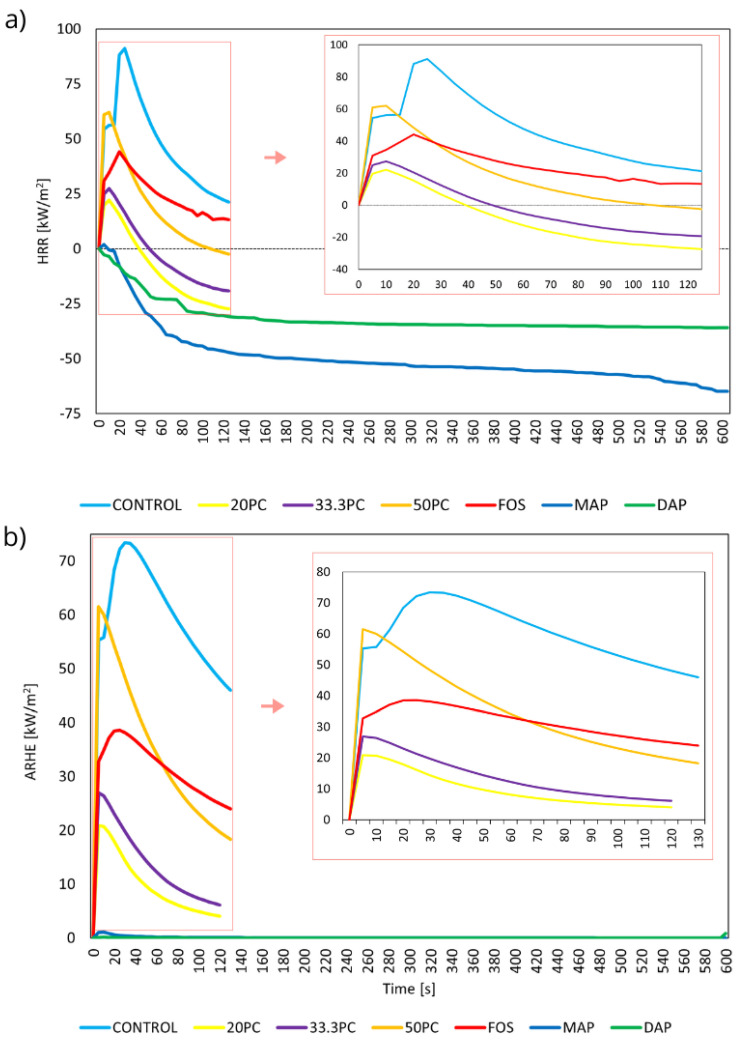
Cone calorimeter test for untreated and protected samples as a function of time: (**a**) heat-release rate and (**b**) average heat-release rate.

During the MLC tests, the refractory properties of the modified samples improved significantly compared to the control samples. All samples were subjected to a heat flux of 35 kW/m^2^ (Figure 2a,b). Tracing the HRR curve, it was easy to observe that the rapidly increasing value at the beginning of the combustion process is typical for thin materials [36,37,38]. The peak heat-release rate [kW/m^2^] and the heat-release rate [kW/m^2^] were directly dependent on the type of modifying agent used. As their content increased compared to the control samples, the PHRR and HRR values decreased (Figure 2a). Compared to unprotected cellulose, analysis of the PHRR values with 42.9% DAP applied showed a reduction in the peak value of about 75%. Similar observations were made toward MAP-protected samples (peak value reduction of about 75%). For the ARHE [kW/m^2^] curve (Figure 2b), the average heat-release rate was highest for the control sample, and the lowest values were recorded for MAP- and DAP-protected samples.

A similar relationship was observed for the THR [MJ/m^2^] curve (Table 3), where the total heat release reached the highest values for the control sample and the lowest values for MAP- and DAP-protected samples. It was also observed that, as the PC concentration increased, the samples’ flame retardancy increased. Each sample treated with PC expanded and created a foamy char layer.

When using MAP and DAP supersaturated solutions, the material initially absorbs heat rather than emitting it. It is possible that there was enough phosphate in the samples, resulting in endothermic decomposition, preventing ignition and leading to a lack of thermal decomposition. It is also possible that the coating mechanism stopped heat transport by forming a non-combustible layer, and the gas mechanism led to gas emissions that inhibited combustion. These mechanisms, combined with supersaturated solutions, after which the precipitation of crystals on the surface of the material was observed, may have been a major barrier to the occurrence of ignition of the cellulosic material [37,38,39,40].

When PC was used, an exothermic reaction and the ignition of the material were initially observed, but as the material was exposed to temperature, a foamed layer formed on the surface that inhibited the combustion process and caused heat uptake rather than emission. This may have been due to the melting of potassium, which absorbed the heat, and the emission of carbon dioxide, which reduced the flammability of the material

Consequently, heat uptake was observed for some variants, which generated negative values.

The MLC measurements show that protecting the cellulose with potassium carbonate had a beneficial effect on its parameters by increasing the combustion temperature, stabilizing the combustion process, and improving the combustion efficiency. Protecting cellulose sheets with diammonium phosphate and monoammonium phosphate benefited its flammability by stabilizing the combustion process and improving combustion efficiency. These effects can be important for environmental protection and regarding process efficiency, where control of the combustion process is crucial.

The values of PHRR, EHC, time to ignition, and time to flame out are shown in Table 3. The average ignition time of the samples protected with 20, 33.3% PC, and FOS was similar to that of control samples. In the samples with 50% PC, MAP, and DAP, it was observed that no ignition occurred for 600s of the measurement duration. The test analyses revealed that the samples protected with MAP, DAP, and 50% potassium carbonate showed the greatest reduction of fire parameters.

The MLC study of mass loss confirmed the observation of the MFT test results. According to the literature, the formation of a charred layer of carbon and the emission of non-combustible gases can quickly cause the phenomenon of self-extinguishment, which appeared and probably prevented the spread of flame during the test [41,42].

#### 3.1.3. Oxygen Index

**Figure 3:**
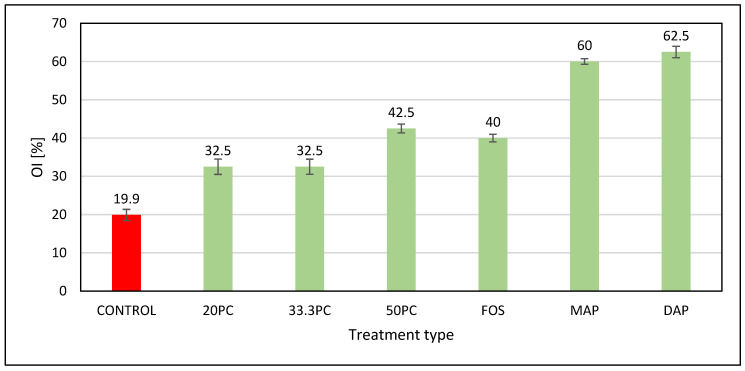
Flammability of the unprotected sample and samples protected by the oxygen index method. The red color indicates combustible variants. The green color indicates non-flammable variants.

It was assumed that obtaining a value of the OI > 25% would allow us to classify the formulations used on the cellulose as flame retardants. Values of OI < 25% consider the tested samples to be flammable [26,27,43]. The properties of the samples, when their flammability was determined by the OI (Figure 3) method, showed that the CONTROL sample was flammable, with an index of 20%. In the case of the protected samples, the index values represented more than 25%, which made it possible to indicate that a lack of flammability characterizes all samples. The highest rate was recorded for DAP and was 62.5%. Thus, the highest effectiveness of this safety measure is once again confirmed.

## 4. Conclusions

Based on the conducted research, it can be concluded that standard flame retardants, commonly found in the construction-materials market, maintain their efficacy when applied to paper products. Cellulose sheets produced using the Rapid Köthen apparatus and subsequently impregnated with aqueous solutions of salts, such as potassium carbonate, monoammonium phosphate, diammonium phosphate, and bisguanidine phosphate, exhibit reduced flammability. The specific flame-retardancy properties of the material vary depending on the type of salt used, as clearly shown in Figure 2a.

Of particular note is the addition of mono- and diammonium phosphate, which through fire testing, facilitated the accelerated charring of modified cellulose fibers and the release of non-flammable gases. This form of modification consequently reduced the combustion temperature and heat release rate, thereby enhancing the flame retardancy of the paper sheets. The cellulose sheets modified with mono- and diammonium phosphate at the 35% (MAP) and 42.9% (DAP) concentrations exhibited the highest level of fire retardancy. In these instances, the samples demonstrated non-flammability, minimal mass loss, and the highest oxygen index values.

It is noteworthy that thermal treatment, such as pressing at a temperature of 220 °C, employed in addition to the Rapid Köthen apparatus, did not compromise the material’s flame-retardant properties. This finding underscores the robustness of the material’s flame retardancy, even under conditions simulating industrial processes in molded-fibers technology.

## Figures and Tables

**Table 1:** The flame-retardant content of each sample based on weight percentage gain (WPG) and dry mass of the retardant.

Treatment Type	WPG [%]	Dry Mass of the Retardant [g/m^2^]
CONTROL	0	0
20PC	11.38 ± 0.13	22.33 ± 1.28
33.3PC	13.54 ± 0.20	29.00 ± 0.82
50PC	14.91 ± 0.12	23.33 ± 0.93
FOS	15.90 ± 0.21	30.00 ± 0.60
MAP	12.19 ± 0.14	20.00 ± 1.18
DAP	14.79 ± 0.33	39.33 ± 1.21

**Table 2:** The maximum value of the exhaust gases temperature for individual samples and time to reach in the MFT method.

Treatment Type	Maximum Temp. of Exhaust Gases (°C)	Time to Reach the Max. Exhaust Gases Temperature (s)	Mass Loss [%]
CONTROL	326.20 ± 46.27	12.00 ± 0.83	99.70 ± 0.32
20PC	119.50 ± 18.61	44.88 ± 16.80	29.44 ± 1.87
33.3PC	122.38 ± 11.54	45.80 ± 11.77	19.07 ± 1.83
50PC	137.28 ± 20.19	54.20 ± 6.58	24.32 ± 4.28
FOS	100.44 ± 12.80	50.20 ± 9.22	17.61 ± 2.84
MAP	156.06 ± 12.79	35.20 ± 5.50	15.30 ± 2.43
DAP	144.83 ± 15.94	55.00 ± 4.97	15.12 ± 2.67

**Table 3:** Maximum values of flammability parameters for the control and treated samples in the MLC test.

Parameters	CONTROL	20PC	33.3PC	50PC	FOS	MAP	DAP
Total Heat Release	5.98 (130 *)	0.49 (60)	0.59 (80)	2.38 (130)	3.12 (130)	0.01 (15)	0.01 (10)
Peak of Heat-Release Rate	56.39 (10 *)	22.15 (5)	28.19 (5)	50.70 (10)	30.10 (10)	11.35 (10)	22.29 (5)
Effective heat of combustion	15.09 (10 *)	9.46 (10)	1.10 (5)	2 (10)	1.58 (10)	0.13 (10)	0 (0)
Time to ignition (s)	6	5	4	X **	6	X	X
Time to flameout (s)	14	12	7	X **	10	X	X

* Time to Maximum Value (s). ** Ignition time not determined; samples did not ignite.

## Data Availability

The data presented in this study are available on request from the corresponding author. The data are not publicly available due to University policies.
